# War Injuries of the Larynx

**Published:** 1918-08

**Authors:** 


### OPHTHALMOLOGY AND OTO-LARYNGOLOGY

**War Injuries of the Larynx.** By W. Douglas Harmer, Surgeon-in-charge of the Throat Department of St. Bartholomew's Hospital. *The Lancet,* June 15, 1918.

The author presents the results of his study of 255 cases of injury to the larynx.

He notes that the commonest place of entry is the anterior triangle of the neck, in the region of the thyroid cartilage. Injuries of the larynx between the vocal cords and the cricoid are the most serious. The pharynx or esophagus is often included. Healing of wounds in the larynx is usually rapid and satisfactory. A considerable proportion develop cheloid or scar-tissue.

Classical symptoms were remarkable by their absence. Cough, dyspnea, blood-spitting, dysphagia, bruising, edema, or inflammation may never occur. Voice is generally lost at once. Hemoptysis is common. Dysphagia is common but transient. Even when the food passage is itself involved, there may be no dysphagia. Injury to the vocal cords is common and paralysis of them from nerve injury or from shock is also frequently observed. Complitions are greatly to be feared in the early stages.

*Treatment* : The first essential is to prevent choking. In doubtful cases tracheotomy should be performed. Nearly a third of the cases required the use of a tube at some time in the course of treatment. Crico-tracheotomy, though easy, is not advisable because the larynx is narrower than the trachea and because the tube if not well tolerated. Inferior tracheotomy is especially liable to complication, and should be practised only when a permanent tube is necessary.

Suture of the air and food passages is the conventional one. To save life partial or total extirpation of the larynx is necessary, because a gangrenous condition of the tissues, if they are badly shattered, often results in death.

Foreign bodies should be removed in all possible cases.

For stenosis a small cannula at first suffices. The patient should wear a tube corked for months before it is finally removed.

There is a comparatively high mortality from wounds of the larynx. More deaths occur at the front than at the base or in England. Of the 108 cases upon which the author chiefly bases his remarks, there were only 5 deaths. Death is commonly due to sepsis, pneumonia, hemorrhage, and dyspnea. The latter are chiefly active in the early stages. In two-thirds of the cases surviving for a week, recovery is complete. In many cases voice weakness persists. Paralysis is another frequent impediment among the unfortunate third that do not recover completely; only a small proportion recover from abductor paralysis. In only a few cases does total paralysis supervene.

The author thinks many of these wounds might be avoided if a band of steel were worn inside the collar to act as a protection similar to that afforded by the helmet.

